# Systems biology research at BGRS-2018

**DOI:** 10.1186/s12918-019-0685-z

**Published:** 2019-03-05

**Authors:** Yuriy L. Orlov, Ralf Hofestädt, Ancha V. Baranova

**Affiliations:** 1 Institute of Cytology and Genetics SB RAS, 630090 Novosibirsk, Russia; 2 Novosibirsk State University, 630090 Novosibirsk, Russia; 3 Bielefeld University, 33615 Bielefeld, Germany; 4 School of Systems Biology, George Mason University, Fairfax, VA USA; 5 Research Centre for Medical Genetics, Moscow, 115478 Russia

In this Special Issue of BMC Systems Biology we present four studies representing systems biology research at BGRS\SB (Bioinformatics of Genome Regulation and Structure\Systems Biology) – 2018 multi-conference (http://conf.bionet.nsc.ru/bgrssb2018/en/). This biannual meeting is conducted in Academgorodok, Novosibirsk since 1998. Other special issues of the BGRS conference series include manuscripts in the fields of plant biology, genomics, bioinformatics and genetics, and collated as BMC Genomics, BMC Evolutionary Biology, BMC Neuroscience, BMC Genetics, BMC Medical Genomics and BMC Plant Biology supplements in recent years [1–4]. We would also like to bring attention of the reader to the highlight of the genetics and bioinformatics research at Belyaev Readings-2017 (http://conf.bionet.nsc.ru/belyaev100/en), which was held in 2017, in BioMed Central journals [5–8].

The papers below were presented in the frames of BGRS\SB–affiliated symposium “Systems Computational Biology” (http://conf.bionet.nsc.ru/bgrssb2018/en/english-systems-computational-biology/) and at 10th International Young Scientists School (SBB-2018) (http://conf.bionet.nsc.ru/bgrssb2018/en/school/).

Dmitry Sakharov and coauthors [9] performed comprehensive comparison of Caco-2 monolayers grown in traditional 2D culture and in a microfluidic chip. It turns out that cultivation conditions influence the spectrum of specific miRNA, secreted by Caco-2 cells substantially. Moreover, these two types of culture display differences in their cell adhesion molecules. This work is an addition to a recent series of papers [10, 11] which describe various steps toward the development of a multi-tissue organs-on-a-chip device.

Alexey Moskalev and co-authors [12] dissected a role of *unpaired 1* gene in ageing of *D. melanogaster* by overexpressing it various organs of the fly*.* In addition to its pronounced and sex-specific effects on a lifespan, overexpressing of *upd1 l*ed to an increase in mRNA levels of the JAK/STAT target genes, pointing that pharmacological modulation of this pathway may eventually help to counteract ageing.

Lyudmila Pastushkova and her colleagues [13] endeavor to perform the study of urine proteome to discover biomarkers of adaptation. Notably, they showed that urine proteome profiles correctly sort individuals into cohorts with one or another predominant type of autonomic regulation of the heart rate. Functional analysis of identified protein biomarkers highlighted many molecules contributing to vascular rigidity. Interested reader should also peruse the previous studies published by the same group, which also investigated the changes in urine proteome detected after spaceflight [14, 15].

Ulyana Zubairova and co-authors [16] developed an ImageJ-plugin for the processing of multi-frame multi-channel 3D images obtained from confocal laser scanning microscopy. This plug-in was extensively tested in high-throughput analysis of cereal leaf epidermis architecture, thus being instrumental for the development of the spatial model for this important plant tissue.

Many authors of the four manuscripts described above are young scientists working on their Master or PhD Dissertation in Biology, Biotechnology or related fields. BioMed Central previously had published special issues comprised of materials prepared by participants of International Young Scientists Schools "Systems Biology and Bioinformatics" (SBB) held in Novosibirsk, Russia (http://conf.bionet.nsc.ru/sbb2018/en/). We invite our readers worldwide to attend our next events - Systems Biology and Bioinformatics Young Scientists School SBB-2019 which will be held in Novosibirsk (http://conf.bionet.nsc.ru/sbb2019/en/), and Vavilov Society Congress - 2019 in St. Petersburg, Russia, in summer 2019. Systems biology field will be highlighted at VII Congress of Vavilov Society of Geneticists and Breeders organized by of Saint Petersburg State University 18–22 June 2019 (https://events.spbu.ru/events/genetic-selection-2019).

## References

[1] Orlov YL, Baranova AV, Hofestadt R, Kolchanov NA (2016). Computational genomics at BGRS\SB-2016: introductory note. BMC Genomics.

[2] Orlov YL, Baranova AV, Salina EA (2016). Computational plant bioscience at BGRS\SB-2016: introductory note. BMC Plant Biol.

[3] Orlov YL, Baranova AV, Markel AL (2016). Computational models in genetics at BGRS\SB-2016: introductory note. BMC Genet.

[4] Orlov YL, Baranova AV, Kolchanov NA, Moroz LL. Evolutionary biology research at BGRS-2018. BMC Evol Biol. 2019;19(Suppl 1). 10.1186/s12862-019-1368-5 10.1186/s12862-019-1368-5 PMC6391745 30813910

[5] Orlov YL, Fernandez-Masso JR, Chen M, Baranova AV. Medical genomics at Belyaev Conference - 2017. BMC Med Genomics. 2018;11(Suppl 1):11. 10.1186/s12920-018-0324-3 PMC5836827 29504904

[6] Orlov YL, Moroz LL, Baranova AV. Neuroscience researches at Belyaev conference-2017. BMC Neurosci. 2018;19(Suppl 1):14. 10.1186/s12868-018-0410-7 PMC5998895 29745834

[7] Orlov YL, Baranova AV, Tatarinova TV, Kolchanov NA (2017). Genetics at Belyaev conference - 2017: introductory note. BMC Genet.

[8] Orlov YL, Baranova AV, Chen M, Salina EA (2017). Plant biology at Belyaev conference - 2017. BMC Plant Biol.

[9] Sakharov DA, Maltseva DV, Knyazev EN, Nikulin SV, Poloznikov AA, Shilin SA, Baranova A, Tsypina IM, Tonevitsky AG. Towards embedding Caco-2 model of gut interface 2 in a microfluidic device to enable multi-organ models for systems biology. BMC Syst Biol. 2019;13(Suppl 1). 10.1186/s12918-019-0686-y. 10.1186/s12918-019-0686-y PMC6399809 30836980

[10] Materne EM, Ramme AP, Terrasso AP, Serra M, Alves PM, Brito C, Sakharov DA, Tonevitsky AG, Lauster R, Marx U (2015). A multi-organ chip co-culture of neurospheres and liver equivalents for long-term substance testing. J Biotechnol.

[11] Poloznikov A, Gazaryan I, Shkurnikov M, Nikulin S, Drapkina O, Baranova A, Tonevitsky A (2018). In vitro and in silico liver models: current trends, challenges and opportunities. ALTEX.

[12] Moskalev A, Proshkina E, Zhavoronkov A, Shaposhnikov M. Effects of *unpaired 1* gene overexpression on the lifespan of *Drosophila melanogaster*. BMC Syst Biol. 2019;13(Suppl 1). 10.1186/s12918-019-0687-x. 10.1186/s12918-019-0687-x PMC6399815 30836998

[13] Pastushkova LH, Rusanov VB, Goncharova AG, Brzhozovskiy AG, Kononikhin AS, Chernikova AG, Kashirina DN, Nosovsky AM, Baevsky RM, Nikolaev EN, Larina IM. Urine proteome changes associated with autonomic regulation of heart rate in cosmonauts. BMC Systems Biol. 2019;13(Suppl 1). 10.1186/s12918-019-0688-9. 10.1186/s12918-019-0688-9 PMC6399814 30836973

[14] Brzhozovskiy A, Kononikhin A, Indeykina M, Pastushkova L, Popov IA, Nikolaev EN, Larina IM (2017). Label-free study of cosmonaut's urinary proteome changes after long-duration spaceflights. Eur J Mass Spectrom (Chichester).

[15] Kononikhin AS, Starodubtseva NL, Pastushkova LK, Kashirina DN, Fedorchenko KY, Brhozovsky AG, Popov IA, Larina IM, Nikolaev EN (2017). Spaceflight induced changes in the human proteome. Expert Rev Proteomics.

[16] Zubairova US, Verman PY, Oshchepkova PA, Elsukova AS, Doroshklov AV. LSM-W^2^: laser scanning microscopy worker for wheat leaf surface morphology. BMC Systems Biol. 2019;13(Suppl 1). 10.1186/s12918-019-0689-8. 10.1186/s12918-019-0689-8 PMC6399813 30836965

